# Smoking cessation interventions and implementations across multiple settings in Japan: a scoping review and supplemental survey

**DOI:** 10.1186/s43058-023-00517-0

**Published:** 2023-11-22

**Authors:** Tomomi Nagasawa, Junko Saito, Miyuki Odawara, Yuki Kaji, Keiichi Yuwaki, Haruhiko Imamura, Kazuya Nogi, Masakazu Nakamura, Taichi Shimazu

**Affiliations:** 1 Department of Health Communication, Graduate School of Medicine, The University of Tokyo, Bunkyo-Ku, Tokyo, Japan; 2 Division of Behavioral Sciences, National Cancer Center Institute for Cancer Control, National Cancer Center, Chuo-Ku, Tokyo, Japan; 3 Department of Cancer Epidemiology, Graduate School of Medicine, The University of Tokyo, Bunkyo-Ku, Tokyo, Japan; 4 Graduate School of Health and Nutrition Sciences, The University of Nagano, Nagano City, Nagano, Japan; 5 Department of Environmental and Occupational Health, School of Medicine, Toho University, Ota-Ku, Tokyo, Japan; 6 Department of Environmental and Occupational Health, Toho University Graduate School of Medicine, Ota-Ku, Tokyo, Japan; 7 Health Promotion Research Center, Institute of Community Medicine, Japan Association for Development of Community Medicine, Chiyoda-Ku, Tokyo, Japan

**Keywords:** Tobacco, Smoking cessation, Implementation science, Japan, Review

## Abstract

**Background:**

Smoking is the leading risk factor for death worldwide. In Japan, although several evidence-based interventions (EBIs) for smoking cessation have been disseminated or adopted, there is a gap between scientific evidence and the actual implementation. This scoping review aimed to describe the knowledge gaps in local-level smoking cessation interventions in Japan, their implementation outcomes, implementation barriers and facilitators, and the use of implementation strategies.

**Methods:**

This study comprised two approaches: (1) a comprehensive scoping review of primary and grey literature, and (2) a supplemental survey of organizations in the grey literature. For the scoping review, we included original studies or reports on smoking cessation interventions targeting adults aged 18 years and older, or providers of cessation support at various settings (community, workplace, school, and clinical settings) in Japan. The extracted data included basic characteristics, intervention categories, implementation outcomes, factors influencing implementation, and implementation strategies for each intervention. Responses to the supplemental survey were extracted same used for the scoping review. To gain a deeper understanding, semi-structured interviews were conducted with some of the organizations in the survey.

**Results:**

A total of 600 interventions with 691 intervention components, based on EBIs in the 2020 US Surgeon General Report, from 498 articles were included in the data extraction; 32 of the 88 organizations responded to the survey. Regarding the overall knowledge about smoking cessation intervention components, behavioral counseling, and cessation medication in clinical settings were mostly reported (34.7%). Implementation outcomes were measured in 18 articles (3.0%) and penetration was mostly reported. Regarding influential factors, “available resources,” and “knowledge and beliefs about the intervention” for barriers, and “relative priority” for facilitators were mostly reported. Implementation strategies were measured in 29 articles (4.8%), and “Train and educate stakeholders” was mostly reported.

**Conclusions:**

Most EBIs reported in the Japanese literature included smoking cessation treatments in clinical settings. While a few articles focused on the implementation indicators in Japan, significant knowledge and experience were extracted from the grey literature, especially in the workplace and community settings. Future research should focus more on implementation to reduce the knowledge gap regarding smoking cessation interventions.

**Supplementary Information:**

The online version contains supplementary material available at 10.1186/s43058-023-00517-0.

Contributions to the literature

- This study contributes to the first synthesis of smoking cessation interventions, implementation outcomes, implementation barriers and facilitators, and implementation strategies for smoking cessation in peer-reviewed articles and a wide range of grey literature in Japan.
- The study results provide practical insights into the implementation of smoking cessation interventions by mapping data using the implementation science framework with intervention components and settings.
- This study proposes a new method for collecting implementation data that combines a scoping review with a quantitative survey and supplements qualitative interviews to gather findings not only revealed in the literature.

## Background

Tobacco use is a major risk factor for cardiovascular and respiratory diseases, and over 20 different types of cancer [1]. In Japan, the smoking prevalence has decreased since 1995. However, the age-adjusted prevalence among adults was 17% in 2019, which is higher than those reported in other developed countries [2].

Evidence of efforts to reduce smoking prevalence is accumulating worldwide [3]. Several EBIs have been systematically implemented in Japan. At an individual level, smoking cessation treatment has been covered by national insurance since 2006 and is available at a copayment of approximately 10–30% of the total fees. In December 2020, smoking cessation treatment apps and carbon monoxide checkers were reimbursed, accelerating online smoking cessation treatments [4]. At a population level, the revised Health Promotion Law went into effect in April 2020, banning smoking indoors in most commercial facilities, restaurants, and offices [5]. While the main purpose of this law revision was to prevent secondhand smoking, it would also reduce smoking prevalence [6]. Regarding approaches for providers, several academic societies have collaborated to establish guidelines for smoking cessation treatments and are working to standardize them [7].

However, the actual implementation of the EBIs is partially limited in Japan. For instance, the number of clinics offering smoking cessation treatment in Japan is approximately 17,000, representing only approximately 15% of all medical facilities, moreover, access is disparate between rural and urban areas [8, 9]. In fact, less than 20% of those who quit smoking used smoking cessation treatment because of a lack of treatment access, lack of media campaigns promoting smoking cessation treatment, and lack of a quitline system [10]. As for smoke-free policies, according to the national survey conducted just one month before the law was fully implemented, approximately 40% of the facilities still allowed indoor smoking [11].

Implementation science aims to promote the adoption and integration of evidence-based interventions into real-world practice [12]. It provides a systematized approach to identifying barriers and facilitators (context of implementation settings), implementation strategies (packages of implementation interventions to address barriers in the context of implementation success), and implementation outcomes (process outcomes of implementation, which indicate implementation success) [13]. To the best of our knowledge, few studies have focused on smoking cessation interventions with an implementation science perspective worldwide, with research being conducted primarily in hospital settings [14–18] and a few in non-hospital settings [19–22], and no reviews have covered settings such as hospitals, communities, workplaces, and schools. Since the implementation of smoking cessation interventions varies globally, and even within countries despite the existence of global and national tobacco control policies [23], there is a need for studies focusing on implementation in a wide range of settings in a specific country.

In Japan, tobacco control measures at a national level are not sufficiently implemented, as only one element reached the best practice in WHO’s MPOWER measures [2]. On the other hand, a grassroots movement for tobacco control, including smoking cessation support, has been implemented widely and contributed to a decrease in smoking prevalence in Japan [24, 25]. The Japanese government also encourages health promotion effort at a local level, including tobacco control measures, in communities and workplaces, and the MHLW recognizes good practices as part of its Smart Life Project [26]. The Ministry of Economy, Trade and Industry (METI) also recommends health promotions for employees from a health management perspective and certifies companies with outstanding efforts [27]. Understanding the reality of smoking cessation interventions and their implementation in these local settings is useful for other countries that have not reached the best practice level in MPOWER because of a lack of national level policies to further promote smoking cessation support.

This study thus aimed to describe the knowledge gaps regarding local-level smoking cessation interventions in Japan, their implementation barriers and facilitators, and the use of implementation strategies. It focused on local-level interventions because national-level interventions require different implementation strategies owing to the scale and variety of stakeholders involved [28]. The research questions (RQs) in this study were as follows:

1. What kinds of smoking cessation interventions (e.g., smoking cessation programs, support, education, and organizational policies) are provided in Japan?
2. What kind of implementation outcomes are evaluated?
3. What are the barriers and facilitators for the implementation of smoking cessation interventions?
4. What are the implementation strategies to promote the use of smoking cessation interventions?

## Methods

In our preliminary screening during the development of the review protocol, we found that the “implementation” of smoking cessation interventions at a local level is limited in primary literature and is more likely to be reported in grey literature, encompassing various document types produced at all levels of the government, academia, businesses, and industries in both print and electronic formats [29]. Scoping reviews involve the synthesis and analysis of a wide range of research and non-research material to provide greater conceptual clarity on a specific topic or field of evidence [30]. Therefore, we decided that a scoping review was the best method for identifying the implementation of smoking cessation interventions in Japan. Furthermore, as information in the grey literature tends to be limited due to reported case studies, we conducted a supplementary survey among the identified organizations reviewed in the grey literature to complement the information regarding the implementation of smoking cessation interventions. Therefore, this study comprised two approaches: (1) a comprehensive scoping review of the primary and grey literature, and (2) a supplemental survey of companies and organizations reported in the grey literature. A detailed description of the methodological steps of this scoping review is provided in our previously published protocol [31].

### Scoping review

The scoping review methodological framework described by Arksey and O'Malley was used [32]. The Preferred Reporting Items for Systematic reviews and Meta-Analyses extension for Scoping Reviews (PRISMA-ScR) published by Tricco et al. as an extension of the PRISMA statement was followed [33], and our findings were reported in accordance with the PRISMA checklist (Additional file 1).

### Literature search strategy and selection criteria

A comprehensive search strategy was reported in the published protocol. The search terms included “tobacco” AND “smoking cessation” OR “health promotion,” in both Japanese and English. The search period was from April 1994, when the first smoking cessation medication was approved in Japan, to September 2022. Searches were conducted in October 2021 for literature from April 1994 to September 2021, and in December 2022 for literature from September 2021 to September 2022 in PubMed, CHINAHL PsycINFO, and Ichushi (a Japanese medical bibliographic database) for peer-reviewed journal articles; and OpenGrey and government reports for grey literature. We reviewed four government sources for grey literature, including (1) Smoking and Health – Report of the Study Group on the Health Effects of Smoking by MHLW [34, 35], (2) the MHLW grants system [36], (3) case studies of the Smart Life Project Award by MHLW [27], and (4) case studies of Health & Productivity Stock Selection Program and the Certified Health & Productivity Management Outstanding Organizations Recognition Program by METI [28], based on consultation with Japanese tobacco researchers. The reference lists of all eligible peer-reviewed journal articles in English were checked for a comprehensive literature search, as suggested by PRISMA-ScR [33].

We included any original studies and reports on smoking cessation interventions targeting adults aged 18 years or older or providers of smoking cessation support. The targeted interventions involved smoking cessation treatment, programs, support, education, and organizational policies aimed at (1) increasing smoking cessation in target populations, primarily focusing on an individual approach; (2) decreasing smoking prevalence in target populations, primarily focusing on a population approach; and (3) developing the skills of individuals engaged in cessation support for adult smokers, primarily focusing on providers. Interventions for the population were included if they were conducted at a local level. Studies that did not primarily focus on tobacco control, did not contain original data, only evaluated drug efficacy, or focused on tobacco control policies at a national or prefectural level were excluded. Studies that did not contain sufficient information about the intervention (studies lacking information on intervention providers, target audience, and intervention settings) were also excluded.

Screening was conducted on two levels. The first screening was performed by reading titles and abstracts, and the second screening was performed by reading full articles. The first screening was conducted by two independent reviewers. The second screening was conducted by one researcher after two independent researchers conducted a pilot test on 10% of the randomly selected articles to confirm understanding of the coding manual among researchers. Conflict in the screening results were discussed at a meeting or via email to reach a consensus. The systematic review approach recommended by Levac et al. was followed for selecting the studies to enhance the rigor of the review [30].

### Data extraction

The first and second authors developed a coding manual and template using Excel for data extraction. The unit of coding included each intervention, defined as a combination of provider, target audience, and intervention settings. We recorded basic study characteristics (author, publication year, country, objectives, characteristics of the study population and its size, and study design) and interventions (type of intervention, intervention component, provider, target populations, settings, duration of intervention, control group, recruitment rate, retention rate, and health outcomes) for RQ1; implementation outcomes (i.e., acceptability, appropriateness, adoption, cost, fidelity, penetration, and sustainability [37]) with detailed description, indicator type, and definition for RQ2; factors influencing implementation (facilitators and barriers) for RQ3; and implementation strategies for RQ4.

We defined EBI components for smoking cessation in accordance with the “sufficient” interventions provided in the Surgeon General Report [4] and added two components of “training and awareness programs to health workers” and “public awareness of tobacco consumption risks and benefits of tobacco cessation” from the WHO Framework Convention on Tobacco Control (FCTC) [38]. The interventions that were not EBIs were classified as “others.” Since a smoking cessation intervention could be multilevel, we allowed the selection of up to two intervention components when coding. For instance, when a workplace intervention consisted of a smoke-free policy at the facility and smoking cessation support for smoking employees, this was coded as one intervention and two intervention components (thus, the number of intervention components can be larger than number of interventions). For RQ1, we did not limit EBIs; however, for RQ2–4, we limited the EBIs for data extraction. Implementation outcome was classified according to Proctor’s definition [37]. For penetration, we included penetration to service recipients and service providers (e.g., the number of eligible persons who use a service, divided by the total number of persons eligible for the service; the number of providers who deliver a given service, divided by the total number of providers expected to deliver the service) [37], as well as penetration to service target organizations (e.g., the number of targeted organizations which apply a service, divided by the total number of organizations expected to apply the service). We made a distinction between penetration to service target organizations and adoption at an organization level, depending on the phase of implementation. Organizational adoption was applied when the study focus was on the early phase of project or policy implementation, and organizational penetration was applied when the study focus was on the current situation. Implementation facilitators and barriers were classified using the consolidated framework for implementation research (CFIR) [39], and the implementation strategies were classified using expert recommendations for implementing change (ERIC) [40, 41]. Components of RQ1, 2, and 4 were extracted from the methods section, whereas the components of RQ4 were extracted from the results section in the literature. To ensure consistency in the interpretation of the classified categories, the first and second authors performed a final check. If necessary, the classified categories were edited based on agreement between the first and second authors.

### Survey

First, we identified municipalities, organizations, and companies conducting smoking cessation interventions by reviewing (3)(4) of the grey literature and mailing questionnaires to health officers of the identified organizations. (i.e., case studies of (3) Smart Life Project Award by MHLW and (4) Health & Productivity Stock Selection Program and the Certified Health & Productivity Management Outstanding Organizations Recognition Program by METI.) The questionnaire focused on identifying facilitators and implementation strategies. Based on a review article and previous literature in Japan, 22 barriers and facilitators were selected from the CFIR constructs, and 26 implementation strategies were selected from ERIC [21, 42–44]. The readability and suitability for the context of the questionnaire items were checked by three health officers implementing health promotion interventions in a Japanese company and a municipal office and revised according to their feedback (see Additional file 2 for detailed question items). The questionnaire survey was conducted in March 2022, and respondents answered either online or on paper. Responses were extracted to the same coding sheet used in the scoping review.

To gain a deeper understanding of the implementation context of the survey responses, qualitative semi-structured interviews were conducted with all organizations that responded that they were available for interviews in the quantitative survey. In the interviews, we aimed to understand the facilitators and implementation strategies that had been mentioned in the cross-sectional survey, including who did what, to whom, when, how often, and what implementation outcomes were aimed for, based on Proctor’s guideline for reporting implementation strategies [45]. Interviews were recorded, and the content was summarized by the interviewers. Qualitative follow-up data were used to complement the authors’ understanding of facilitating factors and implementation strategies in the cross-sectional survey.

The results of both scoping reviews and the survey were summarized in a tabular format, which consists of intervention settings (community, workplace, school, and clinical settings) and intervention components at three levels (provider, population, and individual). We changed the category of hospital setting in our protocol [31] to clinical setting because we included studies in outpatient clinics.

## Results

### Study characteristics

**Fig. 1:**
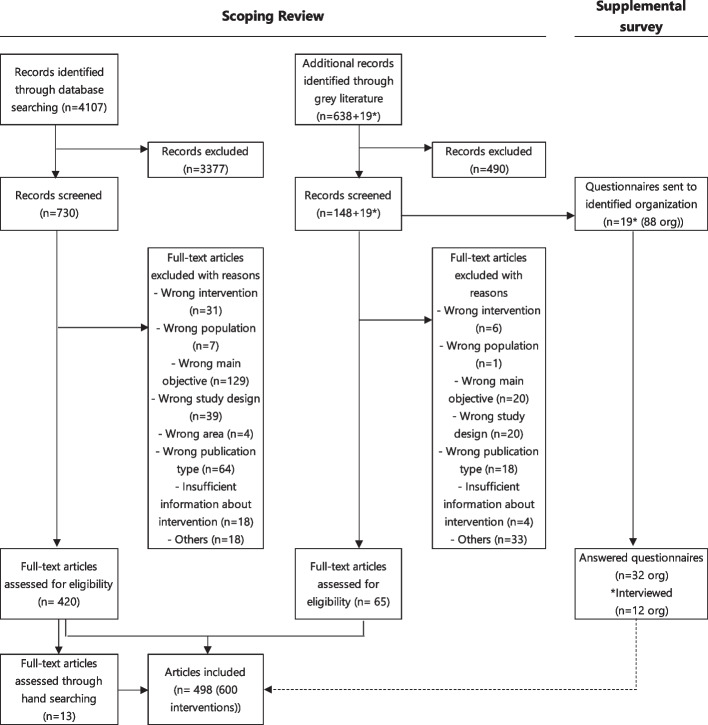
Flow chart. (*The number of gray literature articles (3) case studies of the Smart Life Project Award by MHLW, and (4) case studies of Health & Productivity Stock Selection Program and the Certified Health & Productivity Management Outstanding Organizations Recognition Program by METI is shown separately as they comprise the target of the questionnaire survey)

After removing duplicates, 4,764 records were extracted from the database and grey literature sources. A total of 498 articles were finally included after first screening the title and abstract, and secondly screening the full text, as shown in Fig. 1. The final included 420 reviewed articles (81 of them in English and 339 in Japanese) extracted by a database search, 13 peer-reviewed articles (five of them in English and eight in Japanese) added from a manual search of references of English peer-reviewed articles, and 65 articles through grey literature (46 administrative reports and 19 case studies). After the screening was completed, interventions with different target audiences or settings were categorized separately as different interventions, and 600 interventions from 498 articles were included in the data extraction. Details of the intervention characteristics are shown in Additional file 3 (English peer-reviewed articles only due to volume. Information on other articles is available by contacting the corresponding author).

For the cross-sectional survey, 32 out of 88 organizations identified in the grey literature (19 case studies from MHLW and METI) responded to the survey, 28 of which were businesses, two were municipalities, and two were insurers or non-profit organizations. The role of respondents were public health nurses (*n* = 14), person in charge in the organization (*n* = 13), management (*n* = 2), and others (*n* = 3). Interviews were conducted with 12 organizations, 11 of which were businesses.

### Intervention components and settings

Among the 600 interventions, 691 intervention components were identified and classified by setting, as shown in Table 1. Approaches to providers accounted for 31, approaches to population accounted for 201, and approaches to individuals accounted for 459 of the intervention components, respectively. Behavioral counseling and cessation education accounted for 43.4% of the overall intervention components (300/691 intervention components), while others (interventions not classified as any EBIs) accounted for 19.8% of the same (137/691 intervention components), including smoking cessation advice. EBIs not extracted in this study included quality and performance measures, payment reform, enhancing the technology of electronic health records, quitlines, mass media campaigns, tobacco control programs, proactive behavioral counseling, and short-text message services. By settings, most interventions were conducted in clinical settings (*n* = 328), while the least were conducted in school settings (*n* = 53).

**Table 1:** Intervention components by settings (*n* = 691)

		**Settings of interventions**			
**Interventions components**	**Total**	**Community**	**Workplace**	**School**	**Clinical**
**Approach to providers (total)**	**31 (3, 10, 18)**	**15 (0, 2, 13)**	**4 (1, 1, 2)**	**0**	**12 (2, 7, 3)**
- Clinical practice guidelines	1 (0, 0, 1)	0	0	0	1 (0, 0, 1)
- Quality and performance measures and payment reform	0	0	0	0	0
- Enhancing the technology of electronic health records	0	0	0	0	0
- Training or awareness program to health workers	26 (2, 10, 14)	13 (0, 2, 11)	3 (0, 1, 2)	0	10 (2, 7, 1)
- Others	3 (1, 0, 2)	2 (0, 0, 2)	1 (1, 0, 0)	0	0
**Approach to population (total)**	**201 (20, 86, 95)**	**31 (6, 3, 22)**	**100 (6, 24, 70)**	**33 (3, 28, 2)**	**37 (5, 31, 1)**
- Quitlines	0	0	0	0	0
- Smoke-free policies	117 (15, 58, 44)	8 (4, 1, 3)	62 (5, 17, 40)	16 (1, 15, 0)	31 (5, 25, 1)
- Mass media campaign	1 (0, 0, 1)	1 (0, 0, 1)	0	0	0
-Tobacco control programs	0	0	0	0	0
- Public awareness about tobacco consumption risk and benefits of tobacco cessation	65 (5, 25, 35)	19 (2, 2, 15)	25 (1, 6, 18)	16 (2, 12, 2)	5 (0, 5, 0)
- Others	18 (0, 3, 15)	3 (0, 0, 3)	13 (0, 1, 12)	1 (0, 1, 0)	1 (0, 1, 0)
**Approach to individual adults (total)**	**459 (74, 282, 103)**	**68 (16, 25, 27)**	**92 (14, 23, 55)**	**20 (1, 19, 0)**	**279 (43, 215, 21)**
- Behavioral counseling and cessation medication	300 (53, 203, 44)	41 (12, 13, 16)	40 (12, 13, 15)	11 (0, 11, 0)	208 (29, 166, 13)
- Proactive quitline counseling	0	0	0	0	0
- Short text message services	0	0	0	0	0
- Web or internet-based interventions	22 (6, 2, 14)	7 (3, 1, 3)	11 (0, 1, 10)	0	4 (3, 0, 1)
- Others	137 (15, 77, 45)	20 (1, 11, 8)	41 (2, 9, 30)	9 (1, 8, 0)	67 (11, 49, 7)

Numbers in brackets in each cell indicate the number of English original papers, Japanese original papers, and gray literature

### Implementation outcomes of smoking cessation interventions

Implementation outcomes were measured in only 18 articles (including four intervention studies), which accounted for only 3.0% of the total identified interventions (18/600 interventions) [46–64], as shown in Table 2. The implementation outcomes measured included penetration (seven interventions), adoption (four interventions), fidelity (four interventions), and acceptability (four interventions). For instance, as a measure of penetration, it was reported that when manuals and educational materials were prepared and training was provided for municipal health workers, the proportion of municipalities that provided brief smoking cessation advice to all smokers on the day of the group-specific health check-up increased 2.6 times in five years [56].

**Table 2:** Implementation outcomes

Intervention	Implementation outcomes	Detailed description	Indicator type	Indicator definition	
**Approach to providers**					
Training or awareness program to health workers					
Penetration (3)		・Proportion of comprehensive smoking bans < Clinical > (62†)	PBI	**Numerator:** number of psychiatric hospitals that are implementing comprehensive smoking bans	**Denominator:** total number of psychiatric hospitals that were recruited and completed the questionnaire
		・Proportion of implementation of brief smoking cessation advice < Community > (56‡)	PBI	**Numerator:** number of municipalities that provided brief smoking cessation advice to some or all smoking individuals who took specific health checkups under national health insurance	**Denominator:** total number of municipalities in Osaka prefecture
		・Proportion of referral that doctors in regional core hospitals issued to smoking cessation support center at community < Clinical > (59*)	PBI	**Numerator:** number of referrals that doctors in regional core hospitals issued to smoking cessation support centers at community	**Denominator:** total number of smokers in hospitals that participated in the research in Iwate prefecture
Fidelity (2)		・Proportion of smoking cessation support by frequency of implementation < Clinical > (53*†)	PBI	**Numerator:** number of nurses by frequency of performing each action of smoking cessation support (always/usually, sometimes, rarely/never)	**Denominator:** total number of nurses in hospitals that participated in the research in Osaka
		・Score of skill for smoking cessation support < Workplace > (52*†)	CBI	Total score for the six components of smoking cessation support skill (rage: 0–24)	
**Approach to population**					
Smoke-free policies					
Acceptability (2)		・Proportion of positive perceptions of comprehensive smoking bans < School > ( 48†)	PBI	**Numerator:** number of respondents who answered “yes” to several items regarding their positive perception of the comprehensive smoking bans	**Denominator:** total number of staff and students of one public university and users of university-affiliated hospitals in Aichi Prefecture who completed the questionnaire
		・Proportion of agreement to the comprehensive smoking bans < School > (49†)	PBI	**Numerator:** number of respondents who answered “agree” to the implementation of comprehensive smoking bans	**Denominator:** total number of students of one public university in Saitama Prefecture who completed the questionnaire
Adoption (1)		・Proportion of comprehensive smoking bans < Clinical > (46†)	PBI	**Numerator:** number of participating hospitals that are implementing comprehensive smoking bans	**Denominator:** total number of public or private psychiatric hospitals that completed the questionnaire in Japan
Public awareness about tobacco consumption risk and benefits of tobacco cessation					
Adoption (2)		・Proportion of uptake of several smoking cessation activities < Community > (57)	PBI	**Numerator:** number of municipalities implementing each smoking cessation activity (e.g., comprehensive smoking bans in government offices, providing smoking cessation support as part of the health guidance)	**Denominator:** total number of municipalities in Osaka prefecture
		・Number of smoking cessation activities implemented < Community > (58)	CBI	Number of smoking cessation activities implemented in eight public health centers in Kanagawa prefecture	
**Approach to individual adults**					
Behavioral counseling and cessation medication					
Acceptability (1)		・Perceived acceptability of using tools (smoking cessation cards) < Clinical > (47*†)	QE	Perceived acceptability of using smoking cessation cards among pregnant women and their husbands who smoke at obstetrics and gynecology clinics (qualitative description)	
Adoption (1)		・Number of smoking cessation activities implemented < Community > (58)	CBI	Number of smoking cessation support/programs implemented in eight public health centers in Kanagawa prefecture	
Penetration (4)		・Proportion of smoking cessation sessions conducted in the community < Community > (60)	PBI	**Numerator:** number of health centers offering smoking cessation sessions	**Denominator:** number of health centers that completed the questionnaire among all health centers across Japan
		・Proportion of smoking cessation treatment and sessions conducted in the community < Community > (61)	PBI	**Numerator:** number of hospitals offering smoking cessation treatment and sessions for patients	**Denominator:** number of hospitals with more than 300 beds in Japan that completed the questionnaire
		・Proportion of patients who received smoking cessation support based on the clinical path < Clinical > (50*†)	PBI	**Numerator:** number of patients who received smoking cessation support based on the clinical path in the hospital	**Denominator:** number of smoking patients who scheduled to be admitted to the cancer hospital in Osaka prefecture during a given time period and agreed to use a clinical path
		・Proportion of workplaces implementing smoking cessation programs < Workplace > (54†)	PBI	**Numerator:** number of workplaces implementing smoking cessation programs	**Denominator:** number of Japanese workplaces (excluding schools, hospitals, and government offices) where responding occupational physicians were recruited and responded to the survey
Fidelity (2)		・Proportion of health care workers implementing 5As approaches on a daily basis < Clinical > (63†)	PBI	**Numerator:** number of nursing professionals implementing (“always” or “usually”) each 5A component in their daily practice	**Denominator:** number of nursing professionals who participated in smoking cessation support workshops and responded to the survey in Japan
		・Proportion of health care workers implementing 5As approaches < Clinical > (64†)	PBI	**Numerator:** number of dental hygienists implementing each 5A component in their daily practice	**Denominator:** number of dental hygienists in the Kanto region who were recruited and responded to the survey
Web or internet-based intervention					
Acceptability (1)		・Proportion of participants who perceived ease of participation in health guidance for smoking cessation < Workplace > (51*†)	PBI	**Numerator:** number of respondents who answered that the frequency, duration, and amount of diary of the web-based intervention were appropriate	**Denominator:** number of participants in health guidance for smoking cessation

^*^Intervention study, ‡: longitudinal observation study; unmarked: cross-sectional study

^†^Original research

*PBI*, proportion-based indicator; *CBI*, continuous variable indicator; *QE*, quality evaluation

### Barriers and facilitators for the implementation of smoking cessation interventions

Barriers and facilitators were extracted from twelve articles [46, 63–73]. Barriers were extracted from seven articles on smoke-free policy and behavioral counseling and cessation medications, and all of them were reported in the clinical setting. Identified barriers were similar between the two interventions, and the most frequently reported barriers were providers’ “knowledge and beliefs about the intervention,” “available resources,” and “patient needs and resources.” A barrier unique to specific hospitals included the “relative advantage” for a smoke-free policy in psychiatric wards, which referred to concerns about the impact of depriving patients of the opportunity to smoke on psychiatric symptoms rather than the effects of secondhand smoke prevention and smoking cessation [46].

Facilitators were extracted from four articles on community and workplace settings. In the articles, “cosmopolitanism,” “knowledge and beliefs about the interventions,” and “formally appointed internal implementation leaders” were reported. Facilitators were also extracted from the survey, with 32 organizations responding to an average of 5.2 facilitators [27, 28]. When the three most reported facilitators in the workplace and community were extracted per intervention, they were distributed across all domains (Table 3 and more details in Additional file 4). The most frequently reported facilitators, including survey results, were “relative priority,” followed by “evidence strength and quality,” and “patient needs and resources.”

**Table 3:** Barriers and facilitators for the implementation of smoking cessation interventions

	Consolidated Framework for Implementation Research (CFIR) constructs				
ITV components	ITV level				
	**1. Intervention characteristics**	**2. Outer setting**	**3. Inner setting**	**4. Individual characteristics**	**5. Process**
**Barriers**					
**Approach to population**					
Smoke-free policies	Relative advantage < Clinical > [46]†	Patient needs and resources < Clinical > [46]†		Knowledge and beliefs about the intervention < Clinical > [71†]	
		External policy and incentive < Clinical > [71†]			
**Approach to individual adults**					
Behavioral counseling and cessation medication		Patient needs and resources < Clinical > [70†, 71†, 72†]	Compatibility < Clinical > [70†, 71†]	Knowledge and beliefs about the intervention < Clinical > [66†, 70†, 71†, 72†]	
		External policy and incentive < Clinical > [71†]	Available resources < Clinical > [65†, 69†, 70†, 71†, 72†]	Self-efficacy < Clinical > [70†]	
				Other personal attributes < Community > [66†, 70]	
**Facilitators**					
**Approach to population**					
Smoke-free policies	Evidence strength and quality < Workplace > [27, 28]	Cosmopolitanism < Workplace, School > [73]	Relative priority < Community > [68†] < Workplace > [27, 28]	Knowledge and beliefs about the intervention < Community > [67†]	Formally appointed internal implementation leaders < Workplace, School > [73]
		Patient needs and resources < Workplace > [27, 28]			
		External policy and incentive < Community > [27, 28]			
Public awareness about tobacco consumption risk and benefits of tobacco cessation	Evidence strength and quality < Community > [27, 28]	Cosmopolitanism < Workplace, School > [73]	Relative priority < Workplace > [27, 28] < Community > [27, 28]	Knowledge and beliefs about the intervention < Community > [67†]	Formally appointed internal implementation leaders < Workplace, School > [73]
	Cost < Community > [27, 28]	Patient needs and resources < Workplace > [27, 28]		Individual stage of change < Community > [67†]	
		External policy and incentive < Community > [27,28 ]		Other personal attributes < Community > [67†]	
**Approach to individual adults**					
Behavioral counseling and cessation medication	Evidence strength and quality < Workplace > [27, 28] < Community > [27, 28]	Patient needs and resources < Workplace > [27, 28]	Relative priority < Community > [68†,^27^,^28^] < Workplace > [27, 28]	Knowledge and beliefs about the intervention < Clinical > [63†, 64†] < Community > [67†]	
	Cost < Community > [27, 28]		Access to knowledge and information < Community > [66†]	Self-efficacy < Clinical > [64†] < Community > [66†]	
				Individual stage of change < Community > [67†]	
				Other personal attributes < Community > [67†]	
Web or internet-based intervention	Evidence strength and quality < Workplace > [27, 28]	Patient needs and resources < Workplace > [27, 28]	Relative priority < Workplace > [27, 28]		

^†^Original research, <  > refers to the setting

### Implementation strategies to accelerate smoking cessation interventions

There were 28 articles reporting implementation strategies, accounting for 4.7% of the total identified interventions (28/600 interventions) [51, 56, 74–99]. Nineteen of these were peer-reviewed articles. Implementation strategies were also extracted from the survey, with 32 organizations responding to an average of 11.3 implementation strategies [27, 28]. The three most reported strategies in the workplace and community were extracted per intervention. The most frequently used implementation strategy, including the survey results, was “train and educate stakeholders,” followed by “engage consumers” and “develop stakeholder interrelationships” (Table 4 and more details in Additional file 5). According to the survey results, the most frequently identified strategy was “develop stakeholder interrelationships” in the community (e.g., collaboration between city leaders, city board members, and health advisory committee members regarding tobacco control [example from the interview]) and “adapt and tailor to context” in the workplace (e.g., adjusting measures based on the differences in smoking prevalence and culture among offices and departments, even within the same company [example from the interview]).

**Table 4:** Implementation strategies for smoking cessation interventions

	**Implementation strategies (ERIC)**							
ITV components	ERIC level							
	**A. Use evaluative and iterative strategies**	**C. Adapt and tailor to context**	**D. Develop stakeholder interrelationships**	**E. Train and educate stakeholders**	**F. Support clinicians**	**G. Engage consumers**	**H. Utilize financial strategies**	**I. Change infrastructure**
**Approach for individual adults (total)**								
Smoke-free policies	A-7. Develop and organize quality monitoring systems < Workplace > [88†] A-9. Stage implementation scale up < Workplace > [88†]	C-2. Tailor strategies < Workplace > [27, 28]	D-1. Build a coalition < Clinical > [88†, 89†] < Community > [27, 28] D-12. Organize clinician implementation team meetings < Workplace > [89†] < Clinical > [91†]	E-1. Conduct educational meetings < Workplace > [88†, 97,89†, ] < Workplace > [27, 28] E-3. Conduct ongoing training < Workplace > [27, 28] E-4. Create a learning collaborative < Workplace > [97] E-5. Develop educational materials < Clinical > [95] E-6. Distribute educational materials < Workplace > [88†]		G-3. Involve patients/consumers and family members < Clinical > [91†] G-5. Use mass media < Workplace > [27, 28]	H-2. Alter incentive/allowance structures < Workplace > [88†]	
Public awareness about tobacco consumption risk and benefits of tobacco cessation	A-4. Conduct a local needs assessment < Community > [27, 28]		D-1. Build a coalition < Community > [27, 28]	E-1. Conduct educational meetings < Community > [56] < Workplace > [27, 28] < Community > [27, 28] E-3. Conduct ongoing training < Community > [56] < Workplace > [27, 28] E-5. Develop educational materials < Community > [56] E-6. Distribute educational materials < Workplace > [76†]		G-5. Use mass media < Workplace > [27, 28] < Community > [27, 28]		
**Approach for individual adults**								
Behavioral counseling and cessation medication	A-4. Conduct a local needs assessment < Community > [27, 28] A-6. Develop and implement tools for quality monitoring < Clinical > [85†]	C-2. Tailor strategies < Clinical > [81†] < Workplace > [27, 28]	D-1. Build a coalition < Clinical > [75†] < Community > [27, 28]	E-1. Conduct educational meetings < Clinical > [74, 81,98,75†, ] < Workplace > [94, 97] < Workplace > [27, 28] < Community > [27, 28] E-3. Conduct ongoing training < Clinical > [74, 82] < Community > [79†] < Workplace > [27, 28] E-4. Create a learning collaborative < Workplace > [97] E-5. Develop educational materials < Clinical > [81†, 90†, 93†] E-6. Distribute educational materials < Workplace > [76†, 77†] < Clinical > [78, 98] E-7.Make training dynamic < Clinical > [74†]	F-1. Create new clinical teams < Clinical > [87†] F-2.Develop resource sharing agreements < Clinical > [75†]	G-2. Intervene with patients/consumers to enhance uptake and adherence < Workplace > [80†] < Clinical > [83†, 93,92†, ] < Community > [96] G-3. Involve patients/consumers and family members < Workplace > [80†] G-5. Use mass media < Workplace > [27, 28] < Community > [27, 28]	H-2. Alter incentive/ allowance structures < Workplace > [80†, 86†, 89†] < Clinical > [92†]	I-4. Change record systems < Workplace > [84] < Clinical > [99] I-7. Mandate change < Clinical > [99]
Web or internet-based intervention		C-2. Tailor strategies < Workplace > [27, 28]		E-1. Conduct educational meetings < Workplace > [27, 28] E-3. Conduct ongoing training < Workplace > [27, 28]		G-4. Prepare patients/consumers to be active participants < Workplace > [51†]		

^†^Original research, <  > refers to the setting

The ERIC level “B. Provide interactive assistance” is missing because no information was extracted in this category

## Discussion

This scoping review described the knowledge gaps in local-level smoking cessation interventions in Japan, their implementation outcomes, implementation barriers and facilitators, and the use of implementation strategies. Regarding overall knowledge about the smoking cessation intervention components in Japan (RQ1), behavioral counseling and cessation medication in clinical settings were the most commonly used, and articles for community and workplace settings were quite limited. Workplaces have several advantages in implementing health promotion, including smoking cessation interventions, such as enabling access to a large number of people and encouraging sustained peer support and positive peer pressure, which have strong evidence for increasing the prevalence of smoking cessation [100]. While the number of peer-reviewed articles on interventions in workplaces was limited, much knowledge and experience were extracted from the grey literature and supplemental surveys in workplace settings. Moreover, articles on smoking cessation interventions in Japan did not fully cover the list of EBIs reported by the Surgeon General Report. For instance, there are no studies for quitlines despite of strong evidence. To accelerate the implementation of smoking cessation interventions in Japan, there is a need to improve smoking cessation treatment systems, including online treatment, and to develop and disseminate quitline systems [101].

With regard to implementation outcomes (RQ2), a few studies measured them. When an intervention failed to produce the expected effect, this could be caused by either intervention or implementation failure [37]. Measuring implementation outcomes helps us understand the mechanism of success behind implementing an intervention by understanding the implementation process. For instance, fidelity of behavioral counseling can be collected over different time points from various sources such as medical record review (e.g., whether patients were asked, advised, assessed, assisted, or arranged for follow-up) or qualitative approaches included interviews for clinicians or patients [17]. Furthermore, implementation outcomes are not measured only after the implementation. Some implementation outcomes, such as acceptability, can be measured at the pre-implementation phase to each stage of implementation as it is changeable with experience. Since there are already several established EBIs for smoking cessation, it would be beneficial to focus on implementation success by measuring implementation outcomes. None of the 18 articles extracted in this study cited or reported Proctor’s implementation outcome framework. As variations in terminology reporting limited knowledge synthesis across studies [102], future research should use a common taxonomy with conceptual definitions.

Regarding barriers and facilitators (RQ3), the most frequently reported barriers were “available resources,” “knowledge and beliefs about the intervention,” and “patient needs and resources” in clinical settings. These barriers were consistent with previous studies that reported time limitations, low priority, lack of knowledge among healthcare professionals, and providers’ perception that patients were not interested in smoking cessation as barriers to primary care [103, 104], including a scoping review in South Asian regions [105]. The most frequently reported facilitator was “relative priority.” In Japan, the MHLW and METI started awarding and certifying organizations engaged in health promotion in 2012 and 2014, respectively [27, 28]. In addition, a revised Health Promotion Law was enacted in 2018 to reduce secondhand smoking [6]. Thus, these measures at a national level could contribute to prioritizing smoking cessation interventions for organizations. In our study, “patient needs and resources” were reported as both barriers and facilitators. This may be due to differences in implementation phases and settings. While most of barriers were extracted in the implementation phase in clinical settings (e.g., healthcare providers’ hurdles in providing smoking cessation to smokers), most of the facilitators were extracted in the adoption phase in workplace settings as the triggers for introducing smoking cessation interventions in the workplace (e.g., employee complaints about secondhand smoke exposure among non-smoking employees, or requests for implementing measures to support smoking cessation). Regarding implementation strategies (RQ4), the finding that training was the most frequently utilized strategy for smoking cessation interventions in clinical settings was consistent with a previous study [17]. In addition, our study showed that training was utilized not only for behavioral counseling and cessation medication, but also for smoke-free policies, public awareness, and web-based interventions in all settings.

As EBIs have already been established for smoking cessation, it is important to accelerate their implementation. Thus, research should be conducted on methods to increase the evidence of implementation and context by organizing and accumulating barriers, facilitators, strategies, and outcomes within the implementation science framework. In addition, because a knowledge gap was identified through this study, when considering future research funding priorities, it will be possible to prioritize investment in areas where evidence is lacking, which may lead to the promotion of implementation research.

This study has important implications for smoking cessation support practitioners. The findings can be used as a starting point for practitioners to consider what outcomes to measure, what factors may facilitate or inhibit smoking cessation, and what strategies to utilize when implementing a new smoking cessation intervention. For example, when a company is considering the introduction of a new behavioral counseling and cessation medication program, it may be useful to survey the employees’ needs and modify the strategy according to the actual situation in the office, or to consider not only seminars for smokers but also involve the families of employees.

Since this study suggests that organizations conducting smoking cessation interventions have data or insight of implementation outcomes, barriers, facilitators, and strategies, it is important to create reporting tools for implementation outcomes, CFIR, and ERIC that are easy to understand and use for practitioners, encouraging them to accumulate findings in the field. However, because ERIC adopts a conceptual category of implementation strategies, it may not provide enough information for practitioners to utilize. For example, the strategy of “engage consumer” is a large concept, and its sub-items such as “involve patients/consumers and family members” also lack specifics. In our study, we extracted specific examples of “involve patients/consumers and family members,” such as peer-support smoking cessation programs in which smokers and non-smokers are paired to try to quit smoking and a smoking cessation competition by business location, by reviewing case studies and conducting surveys and interviews. Such specific descriptions may be important for practitioners when considering their actions. Therefore, it would be beneficial to include these specific examples along with reporting in the framework of implementation science. Alternatively, utilizing the ERIC compilation and behavior change technique (BCT) taxonomy [106] could be beneficial for both practitioners and researchers to accumulate evidence.

This study also provides important insight for practitioners in countries with lagging tobacco control measures. Even in countries such as Japan, where tobacco control at a national level is insufficient, it could be effective to promote the use of smoking cessation interventions by strengthening the outer setting (i.e., measures by local government, organizational encouragement of health promotion by certificates) and encouraging individual characteristics (i.e., training health workers). Although the Heath & Productivity Stock Selection and Smart Life Projects implemented in Japan do not specifically focus on smoking cessation, they are likely to increase the priority of smoking cessation measures since they are one of the prerequisites for application to them. In addition, smoking cessation treatment is an important measure that can be implemented even if national policies listed in MPOWER are behind it, and the results of this study suggest that improving provider awareness and knowledge is important, since this is a major implementation barrier.

### Strengths and limitation of this study

In this study, we extracted findings that could not be extracted from peer-reviewed articles alone, by utilizing grey literature and quantitative and qualitative research. In particular, the supplemental quantitative survey was able to uncover many findings, with an average of 5.2 responses extracted per organization for facilitators and 11.3 responses per organization for implementation strategies. The number of facilitators was limited to four; however, by including the survey results, we were able to extract more facilitators. Regarding implementation strategies, although the number of articles was large, some strategies could only be extracted through the survey, suggesting that hidden knowledge has accumulated in the field. Furthermore, the interviews allowed us to understand the context of the strategies and facilitators. Combining reviews with quantitative and qualitative research, as in this study, may be useful in understanding the implementation of EBIs already in place.

This study had several limitations. First, despite our efforts to conduct a comprehensive review, the limited number of organization survey results was reflected due to low response rate. Additionally, since our survey collected one response per organization, responses might have been influenced by the respondent’s role/position/time at the organization. Second, although we developed detailed coding manuals for systematic analysis and coding, the results may be affected by misclassification of coding. Third, while implementation strategies for interventions in specific settings are typically developed based on previously identified barriers and facilitators, in this study, the influential factors and the implementation strategies were identified independently and may not align with each other. Finally, as the study only included smoking cessation interventions conducted in Japan, the results may not be generalizable to other contexts. However, the findings of barriers and facilitators as well as implementation strategies could provide important insights for other countries where tobacco control measures at a national level are lagging, as in Japan.

## Conclusions

This scoping review revealed knowledge gaps regarding local-level smoking cessation interventions in Japan, implementation barriers and facilitators, and the use of implementation strategies. Most EBIs reported in the comprehensively searched Japanese literature involved smoking cessation treatments in clinical settings. While a few articles focused on the implementation of smoking cessation interventions, significant knowledge and experience were extracted from the grey literature, especially in the workplace and community settings. Future research should focus on implementation using an implementation science framework to narrow the knowledge gap regarding smoking cessation interventions in the countries where tobacco control measures at a national level are lagging.

### Supplementary Information

**Additional file 1. **PRISMA Checklist.

**Additional file 2.** Survey Questionnaire.

**Additional file 3. **Table 1 with detail (English literature only).

**Additional file 4. **Table 3 with detail.

**Additional file 5. **Table 4 with detail.

## Data Availability

The datasets used and/or analyzed in this study are available from the corresponding author upon reasonable request.
