# Supplementary material for: Smoking cessation interventions and implementations across multiple settings in Japan: a scoping review and supplemental survey
Source: Implement Sci Commun. 2023 Nov 22;4:146. doi: 10.1186/s43058-023-00517-0 (PMC10666296; doi:10.1186/s43058-023-00517-0)
Supplement: Supplementary file 2 — Additional file 2. Survey Questionnaire. [file 43058_2023_517_MOESM2_ESM.docx]

Additional file 2. Survey Questionnaire

**Survey on smoking cessation support at the “Extend Your Healthy Life Expectancy! Award”**

　This questionnaire is part of a research survey named “Research for Smoking Cessation Support Aimed at Extending Healthy Life Expectancy” (Principal Investigator: Taichi Shimazu), funded by the Science Research Fund of the Ministry of Health, Labor, and Welfare (MHLW). This survey aims to collect good practices of smoking cessation measures (mainly smoking cessation support) in the workplace. Furthermore, this survey aims to collect tips on excellent smoking cessation support implemented in participating company and help promoting smoking cessation support in the workplace in our country. The information collected will be used as aggregate data, in a manner that does not identify the name of the business, for use in the MHLW's Grant-in-Aid for Scientific Research reports, conference presentations, and papers.

The survey takes approximately 10 min to complete. If you wish, we will send you the survey results at a later date. Thank you in advance for your cooperation.

**In the following questionnaire, we would like to ask you about the measures (especially smoking cessation support) for which your company received the MHLW's “Extend Your Healthy Life Expectancy! Award.”**

1. Please provide a summary of the measures taken to support smoking cessation (hereafter referred to as “Measures”) for the “Extend Your Life! Award” (hereinafter referred to as the “Award”).

(Outline of measures, duration of implementation, target audience, etc.)

1. Who **initiated the** introduction of this measure? (multiple answers are allowed.)

- Proprietor
- Health Care Manager
- Union health insurance nurse
- Occupational health nurse
- Industrial physician (exclusive or commissioned)
- Other ( 　　　　　　　)
- I don't know.

1. Please select **all that** apply as **direct reasons (factors) for** the **introduction of** this measure.

Factors in the measures themselves

- The measures seemed to be effective.
- They seemed easy to work on.
- They were feasible to implement in terms of cost.
- Other

(Specify: ) 　　　　　　　　　　　　　　)

Factors other than your company

- Learned about other companies' efforts
- To obtain health management certification from the METI
- There were some benefits or rewards for doing health promotion

(Specify: ) 　　　　　　　　　　　　　　)

- A public health nurse or other professional outside your company approached me to see if I would be interested in implementing countermeasures.

(Specify: ) 　　　　　　　　　　　　　　)

- Other

(Specify: ) 　　　　　　　　　　　　　　)

Factors within your company

- To address the issue of secondhand smoking among employees
- Due to the relatively high smoking rate among employees
- Because health management is positioned as a corporate strategy
- Due to requests from employees regarding measures to support smoking cessation
- Due to the enthusiastic efforts of health care personnel and others
- Because of strong leadership by the management
- Due to leaves of absence or resignations for health reasons by experienced employees
- Experienced an accident on the job due to health reasons, etc.
- As the average age of employees has increased, employee health issues have also increased
- To control employee health care costs
- To make the company attractive to applicants for employment / To secure excellent human resources
- To keep current employees working as long and as healthy as possible
- Other

(Specify: ) 　　　　　　　　　　　　　　)

- Please describe in as much detail as possible the other factors that led to the introduction of this measure.

1. Please select **all** reasons you can think of for the **success of** this measure.
2. **Utilizing Employee Opinion**

- 1-1. surveyed the needs of smokers and/or nonsmokers
- 1-2. surveyed the participants about their impression during and after the project implementation.

1. **Match the company/employee**

- 2-1. measures were implemented in accordance with the culture and climate of the company
- 2-2. measures were implemented in accordance with employees' requests

1. **Collaboration**

- 3-1. consulted a public health nurse or occupational physician or asked for instructions
- 3-2. using examples of other companies' efforts
- 3-3. existing internal committees and organizations were involved in the implementation of the measures (e.g., Health and Safety Committee).
- 3-4. organized a new in-house team to implement the measures (e.g., Smoking Cessation Support Project Team)
- 3-5. asked employees who have successfully quit smoking to share their success stories and solicit

1. **Gain/share knowledge**

- 4-1. personnel in charge attended workshops and study groups on health promotion and smoking
- 4-2. provided the employer and other management with scientifically based (evidence-based) information on health promotion and smoking cessation
- 4-3. provided employees with scientifically based (evidence-based) information on health promotion and smoking cessation

(e.g., smoking cessation seminars and e-learning).

1. **Engaging and motivating employees**

- 5-1. provided some kind of individual support for smoking cessation, such as health-care personnel talking to the respondents
- 5-2. used social networking sites (including in-house only), e-mails, bulletin boards, and handouts to boost anti-smoking measures and support smoking cessation.
- 5-3. involved as many colleagues as possible, there were many colleagues who were willing to help

1. **Use of incentives and assistance programs**

- 6-1. established incentive programs for employees (e.g., rewards and recognition for successful smoking cessation and smoking cessation allowances)
- 6-2. established a cost assistance program for smoking cessation treatment for employees
- 6-3. as a company, applied the “Extend You Healthy Life Expectancy!” Award (and received the award).
- 6-4. planned to apply (or have applied) the Ministry of Economy, Trade, and Industry's Health Management Brand/Health Management Excellent Corporation
  - 6-5. established subsidy programs for companies (e.g., various support programs for measures to prevent secondhand smoking in the workplace by the Ministry of Health, Labor, and Welfare)

(Others(specify): 　　　　　)

1. **Changes in measures and priorities**

- 7-1. established a system that allows employees to leave early, arrive late, or take paid leave to visit a smoking cessation clinic
- 7-2. established a system to prohibit smoking during working hours
- 7-3. removed smoking areas on premises or in the company (in case of tenants, smoking areas are no longer allowed)
- 7-4. the employer or other influential people in the company called on to understand and adhere the measures
- 7-5. the employer and health care manager showed enthusiasm, understanding, and support for the smoking cessation policy

**Other**

- 8-1. Other: Please be as specific as possible.

1. Of the various reasons listed above, what do you think was the **most important key to the success of** this measure? Please select **only one.**

Please indicate by number, e.g., 1-1.

＿＿＿＿＿＿＿＿＿

1. Why did you choose the best key selected in Question 5?

Please be as specific as possible.

1. We would like to ask you about your company and current position.

Your company’s name [ ]

Your Position

- Health Care Manager
- Union health insurance nurse
- Occupational health nurse
- Other ( )

Number of employees in your company

- <50 individuals
- 51–100 individuals
- 101–300 individuals
- 301–1000 individuals
- >1001 individuals

1. Please select one of the following options to indicate whether you would like to receive the aggregate results of the survey.

- I would like to receive this information by e-mail
- I would like to receive the information in writing
- I would like to receive both email and written correspondence
- Do not wish to receive the results

1. We are looking for people willing to cooperate with us in a 30-min interview* regarding this measure. If you are willing to cooperate, please check the box below: Thank you for your cooperation.

- Available to cooperate in interviews

*Interviews will be conducted online via ZOOM and TEAMS. If online interviews are not feasible, they can be conducted via telephone.

If you would like us to send you the aggregate results of this survey, or if you would like to cooperate with the interview mentioned under 9.; please provide your company’s name and contact information (e-mail address).

A representative will contact you soon.

Company’s Name

Department’s Name

Your Name

E-mail address

**This is the end of the survey. Thank you very much for your cooperation.**
